# A Network Pharmacology Approach to Investigate the Anticancer Mechanism and Potential Active Ingredients of *Rheum palmatum* L. Against Lung Cancer *via* Induction of Apoptosis

**DOI:** 10.3389/fphar.2020.528308

**Published:** 2020-11-04

**Authors:** Qing Zhang, Jia Liu, Ruolan Li, Rong Zhao, Mengmeng Zhang, Shujun Wei, Dong Ran, Wei Jin, Chunjie Wu

**Affiliations:** ^1^ School of Pharmacy, Chengdu University of Traditional Chinese Medicine, Chengdu, China; ^2^ Emergency Department, Hospital of Chengdu University of Traditional Chinese Medicine, Chengdu, China

**Keywords:** network pharmacology, traditional Chinese medicine, lung cancer, apoptosis, *Rheum palmatum* L. (Dahuang)

## Abstract

*Rheum palmatum* L. (RPL) is a known traditional herbal medicine with the functions of “*heat-clearing and damp-drying*” in traditional Chinese medicine. Its anti-cancer effect against lung cancer has been confirmed previously, but the related mechanisms and active substances for its action has been little studied. This study adopted the network pharmacology, built the network map of drug ingredients and disease targets (DDN), and discussed the effective components of RPL and its possible mechanisms. All constituents of RPL were collected through database search and literature mining, and the potential active constituents were screened. The inverse pharmacophore matching model was used to predict the targets of active ingredients, and the method was supplemented by database retrieval and literature mining. Compounds-target data were inputted into Cytoscape software to build the DDN of RPL, and functional annotation analysis and pathway enrichment analysis were carried out. Finally, 20 active compounds were screened, which acted on 817 targets. A total of 22,418 lung cancer-related targets were collected, and 761 overlapped with drug targets. By bioinformatics annotation of these overlapping genes, a total of 235 gene ontology (GO) functional annotation analyses and 46 Kyoto Encyclopedia of Genes and Genomes (KEGG) pathways were obtained. It was found that the enrichment of GO and KEGG was associated with apoptosis, suggesting RPL plays an anti-lung cancer role *via* inducing cell apoptosis. Subsequent cell experiment results showed RPL and its active constituents inhibited the proliferation of A549 cells and reduced clone formation rate of A549 cells *via* induction of apoptosis. In this study, the pharmacodynamic basis and mechanism of RPL against lung cancer were studied from the perspective of systematic pharmacology, which would be beneficial for further elucidating the anticancer effect of RPL on lung cancer.

## Introduction

Lung cancer, as a malignant tumor of respiratory system, is also one of the most common tumors worldwide and the leading cause of cancer death in both men and women (Peng et al., 2015; Bray et al., 2018). Although surgery, chemotherapy, radiotherapy and targeted drugs have achieved great progress in the prevention and treatment of lung cancer, the death rate of lung cancer patients is still very high. It is reported that the 5-year survival rate of patients with early stage lung cancer is 45–65%, while the 5-year survival rate of patients with advanced lung cancer is only 14% (Syed et al., 2016). Increasing evidences have suggested that herbal medicines are good resources for finding novel drugs, and previous investigations have also demonstrated that lots of extracts or monomers isolated from herbal medicines have the potentials for prevention or treatment of lung cancers (Ma et al., 2012; Peng et al., 2015). *Rheum palmatum* L. (RPL), belonging to the Polygonaceae family, is a known herbal medicine in traditional Chinese medicine (TCM), and modern pharmacological studies have found that RPL has various pharmacological activities including anti-inflammatory, anti-tumor, bacteriostatic, hemostatic, lipid-lowering, and hypotensive effects (Lee et. al., 2012; Jeong et al., 2017; Park et al., 2018). In addition, a growing number of studies have found that RPL extract and its monomers can induce apoptosis in various human cancer cells (Chiu et al., 2009; Ma et al., 2012). Emodin, which is an important natural constituent in plants of Polygonaceae family, such as *Rheum palmatum*, *Polygonum cuspidatum*, and *Rumex japonicas* can inhibit the proliferation of human lung adenocarcinoma A549 cells (Zhang and Guo, 2011; Peng et al., 2013; Li Z. et al., 2019). Nho et al. found that RPL extract can inhibit breast cancer cell migration, movement, and invasion in a concentration-dependent manner *in vitro* (Nho et al., 2015). In 2008, Su et al. found that emodin, a natural anthraquinone derivative from RPL, can induce apoptosis of human lung adenocarcinoma cells (Su et al., 2005).

In recent years, TCM has been widely accepted as a supplement or alternative medicine for the treatment of diseases due to its good therapeutic effect and low toxic and side effects. However, TCM has the characteristics of multiple components, multiple targets, and multiple pathways, which leads to its unclear therapeutic mechanism and medicinal substance basis, and limits its promotion and development. Network pharmacology can systematically analyze the interaction network of drug components, protein targets, diseases, genes, and other elements, which coincides with the overall concept of TCM treatment. Therefore, the application of network pharmacology to the study of TCM is scientific and necessary. At present, more and more scholars have applied network pharmacology to the research on the material basis and mechanism of action of TCM on various diseases (Jiang et al., 2019; Xiong et al., 2019). In this study, network pharmacology was used to study the active ingredients and mechanism of action of RPL against lung cancer and in combination with *in vitro* experiments to provide scientific evidence for the anti-lung cancer effect of RPL.

## Materials and Methods

### Active Components Screening

All the chemical components of RPL were retrieved from TCM systematic pharmacology database (TCMSP; http://lsp.nwu.edu.cn/tcmsp.php), integrated pharmacology-based research platform of TCM V2.0 (TCMIP; www.tcmip.cn/TCMIP/index.php/Home/Index/All) and TCM integrated pharmacology database (TCMID, https://omictools.com/tcmid-tool), and supplemented by literature mining. Oral bioavailability (OB) is an important parameter to measure the pharmacokinetic process (absorption, distribution, metabolism and excretion) of drugs *in vivo* as well as druggability. Drug-Likeness (DL) refers to the physical and chemical properties related to good clinical efficacy, such as solubility, stability, biological properties, *etc*. Therefore, it is also known as the similarity between the molecule to be tested and the drug molecule, which has a considerable indicator role in the development of new drugs. Compounds with high properties of OB and DL are not drugs but have the potential to become drugs, so these two parameters are often used to screen for active compounds. The compounds of RPL were screened for activity using the TCMSP platform and the DL prediction tool provided by the molsoft website (http://www.molsoft.com/docking.html), and compounds meeting DL ≥ 0.18 and OB ≥30% were selected as candidate active ingredients. Searching for the standard names and specific structures of the active candidate compounds in SciFinder (https://scifinder.cas.org/), PubChem (https://pubchem.ncbi.nlm.nih.gov/), and ChemSpider (http://www.chemspider.com/), and use ChemDraw 14.0 to draw the structures of these compounds, which are stored in MOL format.

### Establishment of Target Library

Information on target proteins of candidate compounds was collected from the TCMSP database and TCMID, and the target proteins of these compounds were obtained by searching Pubmed database. In addition, in order to retrieve the completeness, we also use Pharmmapper online database (http://www.lilab-ecust.cn/pharmmapper/) and Swiss Target Prediction database (http://www.swisstargetprediction.ch/) looking for compound rhubarb targets. In simple terms, the candidate compounds are uploaded to an online database, the species are restricted to human sources, and potential targets are identified through high-throughput screening. These targets were combined and the repeated target sets of small molecular compounds were deleted. Online Mendelian Inheritance in Man (OMIM; https://omim.org/), DisGeNET (http://www.disgenet.org/), TTD (http://db.idrblab.net/ttd/), and GeneCards (https://www.genecards.org/) were used to search the therapeutic targets related to lung cancer, and the repeated targets were removed to construct the target set of the disease. Finally, the two targets were collected by R software and the intersection was taken as the protein target library of this study. Standard names for all protein targets were identified through UniProt (http://www.uniprot.org/).

### Network Construction

Cytoscape is a software that graphically displays the network, analyzes and edits the data, and visualizes it. At the same time, it contains a large number of functional plug-ins for in-depth analysis of the network, adding rich annotation information. Based on the previous steps, we prepared the data pairs of active compounds and disease target genes with R software, imported the data pairs into cytoscape software, and constructed the network map of drug ingredients and disease targets (DDN). In the network diagram, the nodes represent drugs, active ingredients, target genes, and diseases, and the edges represent the interrelationships among the nodes. In addition, the network analyzer plug-in is used to calculate Degree, Closeness, Node-betweenness, K-coreness, Edge-betweenness, and other important parameters to assess the topological properties of each node in the networks in order to make clear the more important composition of RPL and their targets, further scientific and rational interpretation of its molecular mechanisms for the treatment of lung cancer.

### Protein-Protein Interaction Network

The regulation of various biological processes in the body is not regulated by a single protein or gene, but through a complex regulatory network. There is a certain degree of signal transduction between different signaling pathways and targets, so the effective components of drugs are more likely to exert their effects by acting on multiple proteins or multiple signaling pathways. String (https://string-db.org/) is an open source online database that can be used to analyze known and predicted interactions between proteins. The target proteins were uploaded to the string database, and the study species were limited as human sources. Download the protein interaction relationship, save it as TSV format, import it into the Cytoscape software, and construct the protein-protein interaction network diagram (PPI) in which each node represents a protein, and the connection between nodes represents the interaction between two proteins. Meanwhile, cytoHubba tool is used to analyze the core regulatory genes of PPI network.

#### Bioinformatic Annotation

Proteins from the combined target library were uploaded to Funrich or R software for functional annotation analysis and pathway enrichment analysis, including gene ontology (GO) and Kyoto Encyclopedia of Genes and Genomes (KEGG) pathway enrichment analysis. The GO enrichment analysis includes cellular component (CC), biological process (BP), and molecular function (MF), showing 10 remarkably rich terms in each category, with bar charts drawn by Funrich software. The results of KEGG pathway enrichment were used to explain the potential molecular mechanism of RPL for lung cancer and the bar and bubble diagrams of the significant KEGG pathway were drawn by the R language tool. *P* < 0.05 was considered statistically significant in this study.

### Experimental Validation

#### Chemicals and Reagents

Fetal bovine serum (FBS), phosphate buffered saline (PBS), penicillin-streptomycin, trypsin-EDTA and dulbecco modified eagle medium (DMEM) were purchased from GIBCO (Grand Island, NY, USA). Dimethyl sulfoxide (DMSO) was provided by Sigma-Aldrich Co. (St. Louis, MO, USA). The 4 ,6-diamidino-2- phenylindole (DAPI) and Annexin V-FITC/PI apoptosis kits were obtained from the US Everbright® Inc (Suzhou, China). Cell Counting Kit-8 (CCK8) detection kit was obtained from the Beijing 4A Biotech Co., Ltd (Beijing, China). Annexin V-FITC/PI apoptosis kits were purchased from BOSTER biological technology company (Wuhan, China). The paraformaldehyde and crystal violet staining solution were obtained from Chengdu Kelong Chemical reagent factory (Chengdu, China). All the used reference substances were purchased from the PUSH Bio-Technology Co. (https://www.push-herbchem.com/, Chengdu, China) with the purity over 98%.

#### Preparation of the Lyophilized Powder of RPL Extracts

The herbal medicine was purchased from the Neautus Chinese Herbal Pieces Ltd. Co. (Chengdu, China), and identified by Prof. Chunjie Wu (School of Pharmacy, Chengdu University of TCM). A voucher specimen for future reference has been deposited in Department of Chinese Medicinal Processing, School of Pharmacy, Chengdu University of TCM, Chengdu, China (no. 2019091701#).

The crude herbal medicine (126 g) was powered and refluxed with eight times 70% ethanol (1:8, w/v) for 30 min, then the filtrates were concentrated, and the ethanol was recovered using a rotary evaporator, subsequently the extracts were freeze-dried by a LGJ-12B freeze dryer (Shanghai GIPP Co. Ltd, Shanghai, China). Furthermore, the HPLC assay of the RPL extracts was carried out, and the results showed the main constituents in RPL are anthraquinones such as Sennoside A, B, Rhein and Aloe-emodin and its glucoside, emodin and its glucoside, Chrysophanol and its glucoside, etc (**Figure S1**).

#### Cell Culture

Human lung cancer cell line A549 and human umbilical vein endothelial cells (HUVEC) were purchased from the Beina Biological Co. (Beijing, China), and cultured at 37°C in a humidified atmosphere of 5% CO and 95% air in sterile DMEM medium with 10% FBS and supplemented with 100 U/mL penicillin, 100 U/mL streptomycin.

#### Cell Activity Detection

CCK-8 assays were carried out to detect the effect of RPL on A549 cell and HUVEC cell activity. Briefly, the cells were seeded into 96-well plate (1×10^4^/well) and cultured for 12h at 37°C. After that, cells were treated with different does of RPL for 24h in a 5% CO_2_ incubator at 37°C, 10 µL CCK-8 was added to each well and incubated for 1h. The optical density (OD) values were measured at 450 nm using a microtablet reader (Bio-RAD, USA). Each experiment was repeated three times individually.

#### Plate Clone Formation Assay

Cells were seeded in 6 well plates with 1000 cells per well for 24 h of drug intervention (RPL 0.2, 0.4, and 0.6 mg/mL). Continue incubation for 14 days, then wash twice with PBS, and stained with Crystal Violet Staining (Sangon Biotech, China). The colony formation was observed under a microscope, and five fields were randomly selected for counting. Each experiment was repeated three times.

#### DAPI Staining

DAPI staining was used to determine whether the activity inhibition of RPL on A549 cells was related to apoptosis induction in accordance with previous reported methods. The cells were incubated with different concentrations of RPL (0.2, 0.4, and 0.6 mg/mL) for 24 h and fixed with 4% paraformaldehyde at room temperature for 15 min. The cells were then stained with DAPI solution at room temperature for 10 min and washed in PBS, and the image data were observed and recorded under an Olympus IX71 inverted fluorescence microscopy (Olympus, Tokyo, Japan). Each experiment was repeated three times.

#### Apoptosis Assay by Flow Cytometer

Cell apoptosis was detected by flow cytometry (CytoFLEX FCM, Beckman Coulter Inc., Atlanta, Georgia, USA). The A549 cells were seeded on six-well plates for 12 h and treated with different concentrations of RPL (0.2, 0.4, and 0.6 mg/mL) for 24 h. After that, the cells were collected, washed, and centrifuged. According to the manufacturer’s instructions of Annexin V/PI apoptosis kit, the cells were resuscitated with 500 μL of binding buffer, mixed with 5μL of Annexin V-FITC and PI, and incubated at room temperature in dark for 15 min. The cell apoptosis was detected by ﬂow cytometry analysis. Each experiment was repeated three times.

#### Statistical Analysis

Results were expressed in terms of mean and standard deviation (SD), and all statistical comparisons were evaluated using one-way analysis of variance (ANOVA) and significant differences between the mean values were measured using the Duncan’s multiple-range tests.

*P* < 0.01 means the difference is statistically significant.

## Results

### Screening of Active Components

A total of 1380 compounds in RPL were found through database and literature searching. According to the screening results of OB and DL, the OB and DL values of 16 compounds were considered to be “Qualified”, indicating that these 16 compounds had good proprietary properties and were potential active ingredients of RPL in the treatment of lung cancer. Furthermore, some important monmers with low properties of OB and OL in RPL which were reported to possess anti-lung cancer tumors were also included in this study, such as the emodin, resveratrol, chrysophanol, and physcion. At the end of, we obtained 20 potential active ingredients in the RPL, including one terpenoids (Mutatochrome), two steroids (Daucosterol, β-sitosterol), four flavonoids (Eupatin, Catechin, Procyanidin B-5,3’-O-gallate, gallic acid-3-O-(6’-O-galloyl)-glucoside), 13 anthraquinones (Toralactone, Torachrysone-8-O-β-D-(6’-oxayl)-glucoside, Sennoside E, Sennoside D, Rhein, Physciondiglucoside, Palmidin A, Emodin-1-O-β-D-glucopyranoside, Aloe-emodin, emodin, resveratrol, chrysophanol, and physcion), and the detail information of these compounds is listed in **Table 1**.

**Table 1:** The main molecular descriptors for the potential active components.

ID	Compound name	Molecule ID	PubChem CID	MW	AlogP	OB	DL
C1	Toralactone	MOL002281	5321980	272.27	2.25	46.46	0.24
C2	Torachrysone-8-O-β-D-(6’-oxayl)-glucoside	MOL002280	5321979	480.46	0.64	43.02	0.74
C3	Sennoside E	MOL002276	NA	524.5	3.91	50.69	0.61
C4	Sennoside D	MOL002293	NA	524.5	3.91	61.06	0.61
C5	Rhein	MOL002268	10168	284.23	1.88	47.07	0.28
C6	Procyanidin B-5,3’-O-gallate	MOL002260	NA	730.67	4.6	31.99	0.32
C7	Physciondiglucoside	MOL002259	442762	608.6	-0.91	41.65	0.63
C8	palmidin A	MOL002303	5320384	510.52	4.52	32.45	0.65
C9	Mutatochrome	MOL002251	5281246	552.96	10.9	48.64	0.61
C10	gallic acid-3-O-(6’-O-galloyl)-glucoside	MOL000554	11972353	484.4	-0.03	30.25	0.67
C11	Eupatin	MOL002235	5317287	360.34	1.99	50.8	0.41
C12	Emodin-1-O-β-D-glucopyranoside	MOL002288	11968447	432.41	0.59	44.81	0.8
C13	Daucosterol	MOL002297	NA	386.73	7.67	35.89	0.7
C14	β-sitosterol	MOL000359	12303645	414.79	8.08	36.91	0.75
C15	Aloe-emodin	MOL000471	10207	270.25	1.67	83.38	0.24
C16	(-)-catechin	MOL000096	73160	290.29	1.92	49.68	0.24
C17	Emodin	MOL000472	3220	270.25	2.49	24.40	0.24
C18	Resveratrol	MOL012744	445154	228.26	3.01	19.07	0.11
C19	Chrysophanol	MOL001729	10208	254.25	2.76	18.64	0.21
C20	Physcion	MOL000476	10639	284.28	2,74	22.29	0.27

### Screening of Potential Targets

**Figure 1:**
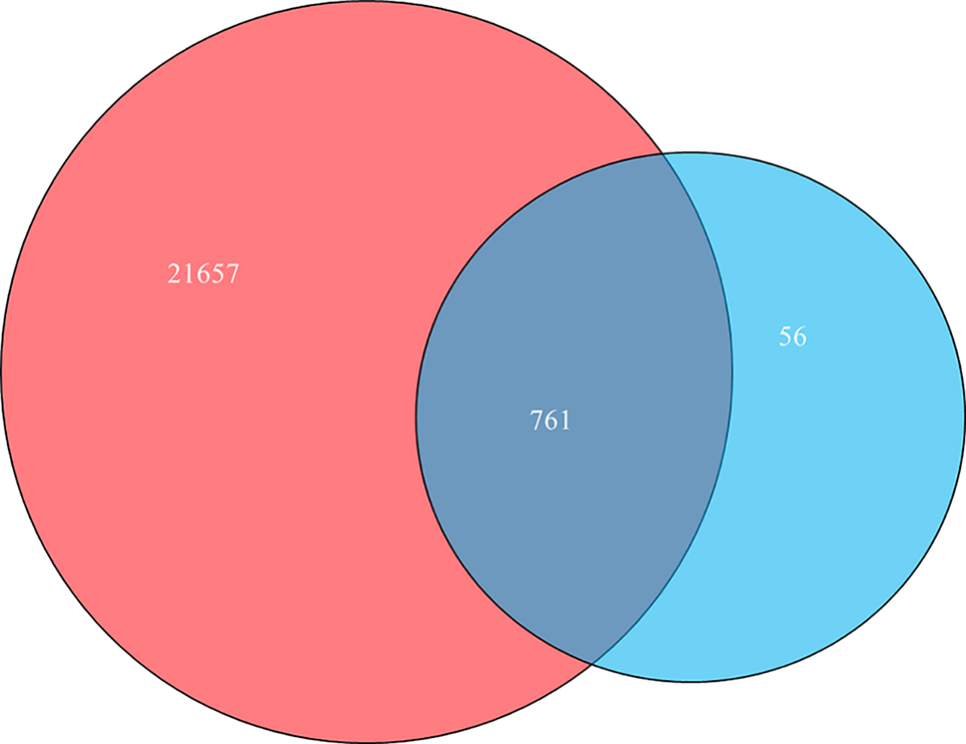
Venn diagram of the potential anti-lung cancer targets.

A total of 22,418 targets related to lung cancer were obtained from OMIM, DisGeNET, TTD, and GeneCards database, and a total of 817 potential targets of 20 active compounds of RPL were collected. As shown in the Venn diagram in **Figure 1**, a total of 761 potential anti-lung cancer targets were obtained through a combined collection of common targets. Part of the target information is shown in **Table 2**. Interestingly, most of the targets of the 20 active compounds in RPL are contained in these 761 targets.

**Table 2:** Degree of core regulatory genes analyzed by Cytoscape.

Name	Symbol	Degree	Average Shortest PathLength		Betweenness Centrality	Closeness Centrality	Neighborhood Connectivity
Insulin	INS	291	1.64760638		0.06648305	0.60694108	60.62199313
RAC-alpha serine/threonine-protein kinase	AKT1	275	1.66888298		0.04719193	0.59920319	65.13454545
Cellular tumor antigen p53	TP53	269	1.67952128		0.05452844	0.59540776	64.11152416
Serum albumin	ALB	258	1.70611702		0.05336222	0.58612627	62.71705426
Interleukin-6	IL6	229	1.76329787		0.02780752	0.56711916	66.6419214
Epidermal growth factor receptor	EGFR	223	1.75664894		0.02834682	0.56926571	70.00896861
Vascular endothelial growth factor A	VEGFA	216	1.7712766		0.01954464	0.56456456	71.61574074
Myc proto-oncogene protein	MYC	207	1.76994681		0.02037922	0.56498873	73.23671498
Proto-oncogene tyrosine-protein kinase Src	SRC	203	1.80585106		0.02519928	0.55375552	71.49261084
Tumor necrosis factor	TNF	201	1.80452128		0.02042326	0.5541636	69.76119403
Caspase-3	CASP3	197	1.80053191		0.01560464	0.55539143	75.9035533
Heat shock protein HSP 90-alpha	HSP90AA1	192	1.79920213		0.02821964	0.55580192	70.609375
Signal transducer and activator of transcription 3	STAT3	175	1.84973404		0.01091074	0.54061826	78.70857143
Estrogen receptor	ESR1	174	1.83909574		0.02209485	0.54374548	76.94252874
Mitogen-activated protein kinase 8	MAPK8	172	1.84308511		0.00999066	0.54256854	79.03488372
Catenin beta-1	CTNNB1	162	1.86835106		0.01984121	0.53523132	74.83950617
Serine/threonine-protein kinase mTOR	MTOR	159	1.86303191		0.00875725	0.53675946	82.40880503
G1/S-specific cyclin-D1	CCND1	155	1.87898936		0.00860106	0.53220099	83.10322581
Receptor tyrosine-protein kinase erbB-2	ERBB2	154	1.86702128		0.00959932	0.53561254	79.12987013
Amyloid beta A4 precursor protein-binding family A member 3	APP	144	1.91888298		0.02165668	0.52113652	66.77777778

### PPI and Hub Genes of RPL Against Lung Cancer

**Figure 2:**
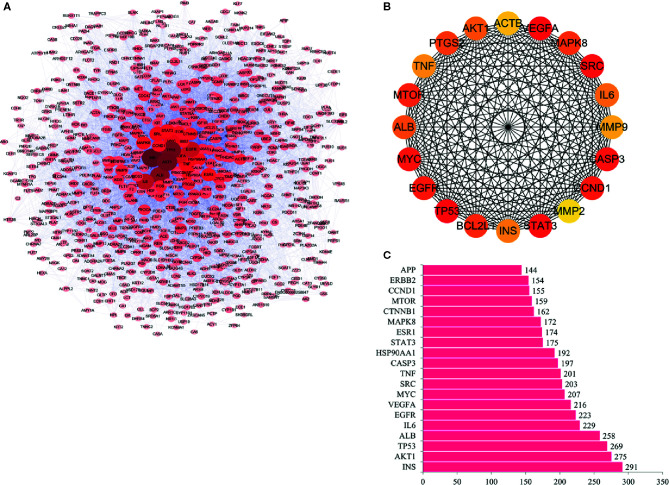
PPI and hub genes of RPL against lung cancer. **(A)** Interaction between proteins. **(B)** Interaction between these genes. **(C)** Genes with highest node degrees.

According to the predictions results of the String, and with the help of cytoscape software, the interaction between proteins was visualized, including 761 nodes and 6840 edge (**Figure 2A**). The top 20 hub genes were screened out according to the degree of nodes, including INS, AKT1, TP53, ALB, IL6, EGFR, VEGFA, MYC, SRC, TNF, CASP3, HSP90AA1, STAT3, ESR1, MAPK8, CTNNB1, MTOR, CCND1, ERBB2, and APP. The interaction between these genes is shown in **Figure 2B**. Among these genes, INS, AKT1,TP53 and ALB have the highest node degrees, which are 291, 275, 269, and 258, respectively (**Figure 2C** and **Table 2**). It is suggested that INS, AKT1, TP53, and ALB may be three key targets for anti-cancer activity of RPL for lung cancer.

### Network Analysis of Targets

**Figure 3:**
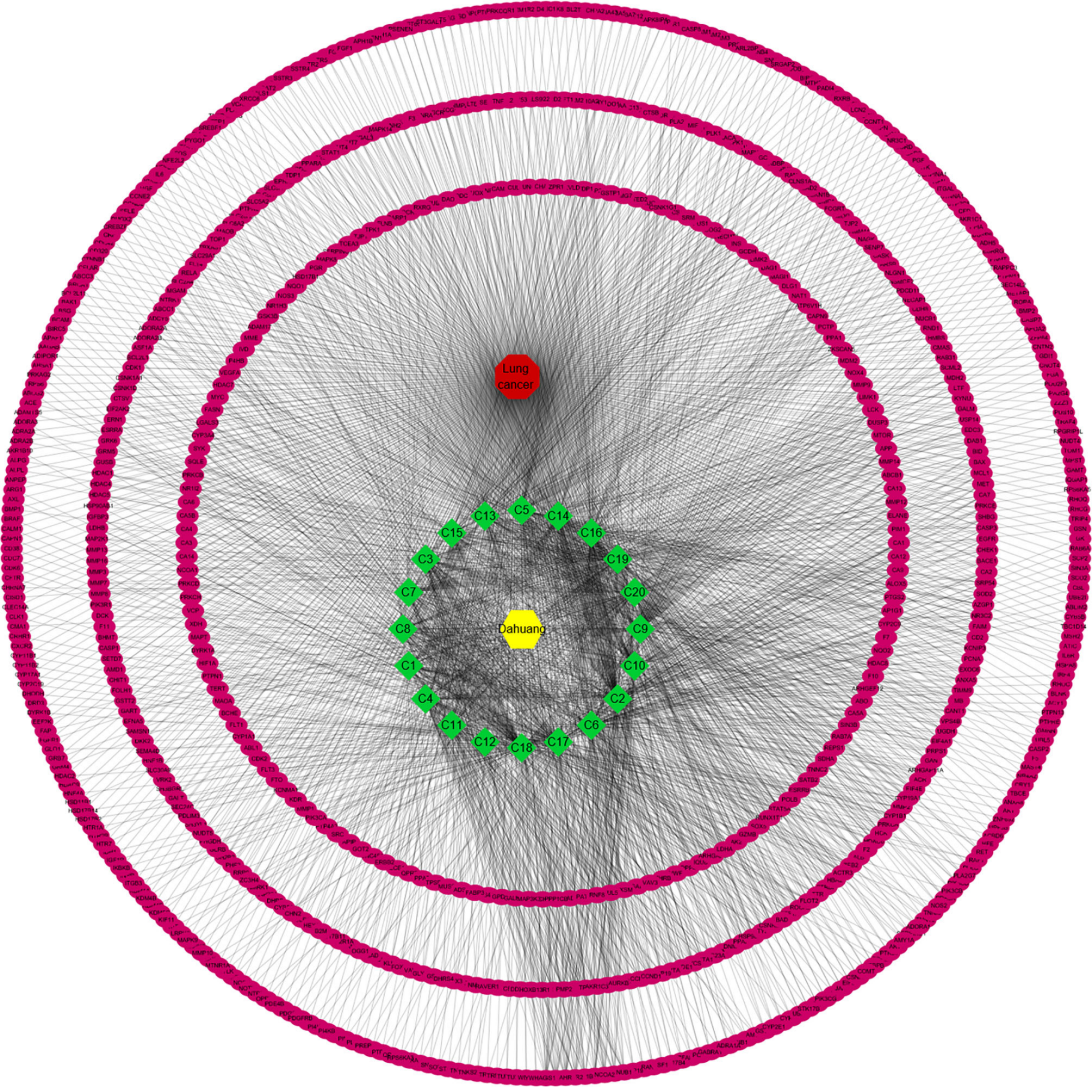
Network analysis of targets. A total of data pairs of active compounds and disease target genes were prepared by R software, and the DDN was constructed.

A total of data pairs of active compounds and disease target genes were prepared by R software, and the DDN constructed is shown in **Figure 3**. This network contains 783 nodes (761 genes, 20 chemicals, 1 drug, and 1 disease) and 2047 edges. In this network, the yellow and red hexagons represent drug and disease, the magenta circle is the protein target, and the green red diamond is the compound. The degree of a node is the number of edges connected to the node, and the higher the degree, the more nodes in the network are directly related to the node, indicating that the node is more important in the network. According to our results, C18, C17, C6, C2, C10, C9, C20, C19, and C16 are linked to more than 100 genes, which are considered to be the main active components for RPL against lung cancer (**Table 3**).

**Table 3:** Degree of 16 active components analyzed by Cytoscape.

Name	Degree	AverageShortestPathLength	BetweennessCentrality	ClosenessCentrality	NeighborhoodConnectivity
C18	178	2.5390525	0.02319971	0.39384771	4.02352941
C17	117	2.68245839	0.00987261	0.37279236	4.5
C6	117	2.67989757	0.00976497	0.37314859	5.47826087
C2	114	2.60947503	0.00911454	0.38321884	4.9380531
C10	111	2.69270166	0.00824008	0.37137423	4.6
C9	110	2.69270166	0.01003758	0.37137423	4.10909091
C20	109	2.6978233	0.00836353	0.3706692	4.2962963
C19	107	2.70038412	0.00822794	0.37031769	4.44859813
C16	100	2.7234315	0.00714986	0.36718383	5.28571429
C14	84	2.75928297	0.00520247	0.36241299	4.85714286
C5	82	2.76696543	0.00482567	0.36140676	5.20987654
C15	79	2.77464789	0.00443887	0.36040609	5.1025641
C13	79	2.77208707	0.00428062	0.36073903	5.20253165
C3	71	2.79257362	0.00360904	0.35809262	5.92957746
C7	68	2.66709347	0.00362509	0.37493999	6.86764706
C8	66	2.80537772	0.00272869	0.35645824	5.90909091
C1	65	2.80793854	0.00300304	0.35613315	5.58461538
C4	63	2.81306018	0.00258953	0.35548475	6.3968254
C11	59	2.82330346	0.00263375	0.35419501	5.06779661
C12	9	2.95134443	0.00002801	0.33882863	10.66666667

### GO Functional Enrichment Analysis

**Figure 4:**
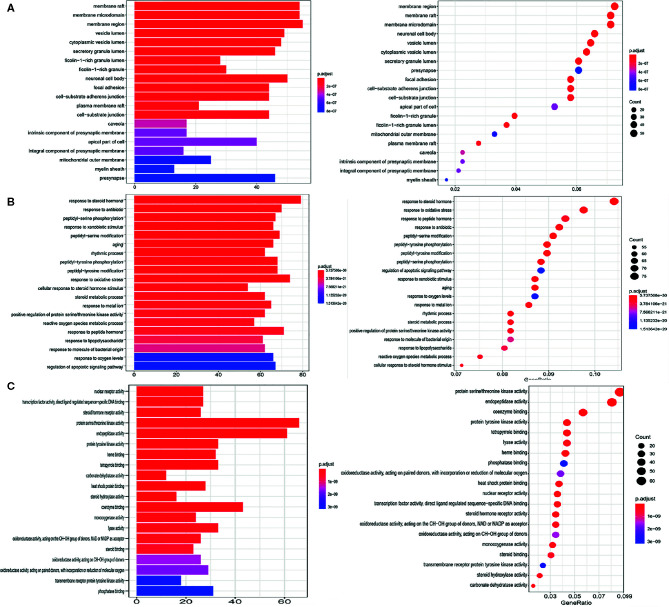
GO functional enrichment analysis. **(A)** Cellular component category. **(B)** Biological process category. **(C)** Molecular function category.

Three types of GO functional annotation analyses were performed for these potential target genes, and the cellular component (CC), biological process (BP) and molecular function (MF) were included. The analysis results as shown in **Figure 4**, the images show only the top 20 related functions. The enriched GO functions for target genes included the membrane raft, membrane microdomain, membrane region, vesicle lumen and cytoplasmic vesicle lumen in the CC category (**Figure 4A**); response to steroid hormone, response to antibiotic, response to oxidative stress, reactive oxygen species metabolic process and regulation of apoptotic signaling pathway in the BP category (**Figure 4B**); kinase activity, endopeptidase activity, protein tyrosine kinase activity and heme binding in the MF category (**Figure 4C**).

### KEGG Pathway Enrichment Analysis

**Figure 5:**
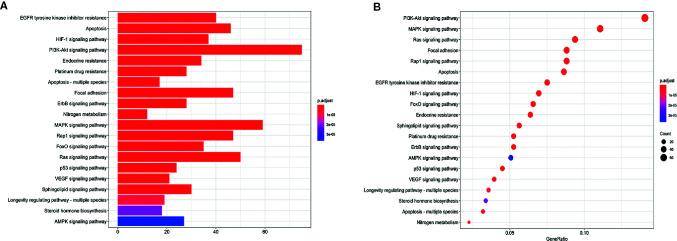
**(A)** Histogram of KEGG enrichment results; **(B)** Bubble diagram of KEGG enrichment results.

The enrichment analysis of the KEGG pathway involved in these target genes was carried out by R language software. The results showed that a total of 533 genes were involved in the enrichment, and 46 pathways significantly correlated with target genes were finally obtained (P ≤ 0.05). The top 20 pathways are shown in **Figure 5** and **Table 4**, mainly including EGFR tyrosine kinase inhibitor resistance, Apoptosis, PI3K-Akt signaling pathway, Apoptosis-multiple species, MAPK signaling pathway, and p53 signaling pathway. Through extensive literature search, these signaling pathways are directly or indirectly related to the occurrence and development of lung cancer, suggesting that these signaling pathways may be closely related to the mechanism of anticancer effect of RPL in lung cancer.

**Table 4:** KEGG pathway enrichment analysis.

ID	Description	P value	P.adjust	Q value	Count
hsa01521	EGFR tyrosine kinase inhibitor resistance	5.14E-22	8.28E-20	4.87E-20	40
hsa04210	Apoptosis	1.89E-16	1.52E-14	8.95E-15	46
hsa04066	HIF-1 signaling pathway	1.68E-13	8.77E-12	5.16E-12	37
hsa04151	PI3K-Akt signaling pathway	2.18E-13	8.77E-12	5.16E-12	75
hsa01522	Endocrine resistance	8.16E-13	2.63E-11	1.55E-11	34
hsa01524	Platinum drug resistance	5.45E-12	1.46E-10	8.60E-11	28
hsa04215	Apoptosis - multiple species	1.78E-10	4.09E-09	2.40E-09	17
hsa04510	Focal adhesion	2.11E-10	4.24E-09	2.50E-09	47
hsa04012	ErbB signaling pathway	3.62E-10	6.48E-09	3.82E-09	28
hsa00910	Nitrogen metabolism	9.18E-10	1.48E-08	8.69E-09	12
hsa04010	MAPK signaling pathway	1.16E-09	1.70E-08	1.00E-08	59
hsa04015	Rap1 signaling pathway	1.45E-09	1.81E-08	1.07E-08	47
hsa04068	FoxO signaling pathway	1.46E-09	1.81E-08	1.07E-08	35
hsa04014	Ras signaling pathway	1.69E-09	1.94E-08	1.14E-08	50
hsa04115	p53 signaling pathway	5.16E-09	5.54E-08	3.26E-08	24
hsa04370	VEGF signaling pathway	1.26E-08	1.27E-07	7.46E-08	21
hsa04071	Sphingolipid signaling pathway	9.42E-08	8.92E-07	5.25E-07	30
hsa04213	Longevity regulating pathway - multiple species	9.21E-07	8.24E-06	4.85E-06	19
hsa00140	Steroid hormone biosynthesis	2.52E-06	2.14E-05	1.26E-05	18
hsa04152	AMPK signaling pathway	4.54E-06	3.66E-05	2.15E-05	27

**Figure 6:**
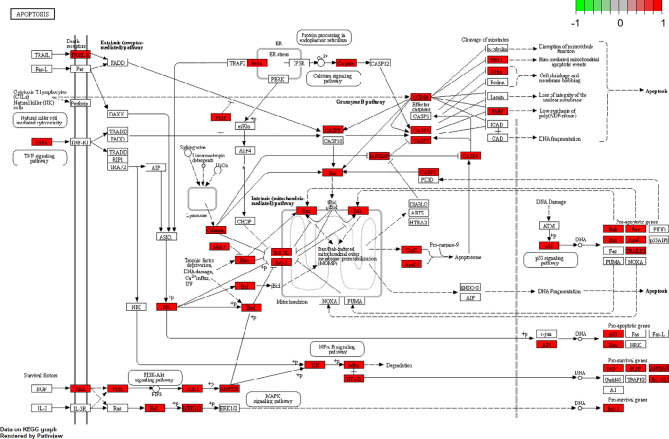
The potential apoptotic pathway for anticancer effects of RPL against lung cancer.

More and more evidence shows that the abnormal regulation of apoptosis gene plays an important role in the occurrence and development of cancer. Therefore, it is generally accepted that inducing apoptosis of tumor cells is a feasible and most direct treatment for cancer. Interestingly, consistent with the results of the previous GO functional annotation, the KEGG pathway also enriched the apoptotic pathways, suggesting that RPL may play an anti-lung cancer role by inducing apoptosis in lung cancer cells (**Figure 6**). Based on the results of these predicted molecular mechanisms and network analyses, we designed experiments to test our hypothesis at the cellular level.

### RPL Inhibits the Proliferation of A549 Cell

**Figure 7:**
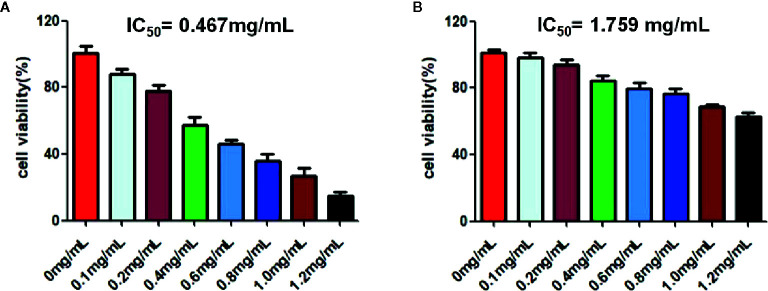
Cytotoxic effects of RPL on A549 cells **(A)** and HUVEC cells **(B)**. Cells were treated with RPL (0, 0.75, 1.5, 3.0, 6.0, 12, and 24 µg/mL) for 24 h. Finally, CCK-8 assays were carried out to detect the cytotoxic effects of candidate drugs. The IC_50_ values were calculated for evauation of the cytotoxic effects of candidate drugs.

CCK-8 was used to investigate the effect of RPL on the activity of A549 cells. As can be seen from **Figure 7A**, RPL at series concentrations (0–1.2 mg/mL) showed the ability to inhibit the proliferation of A549 cells with an obvious concentration-dependent manner. More importantly, RPL did not significantly inhibit HUVEC cells, suggesting that it did not seem to have a significant effect on the viability of normal cells (**Figure 7B**).

### Cloning Formation Assay

**Figure 8:**
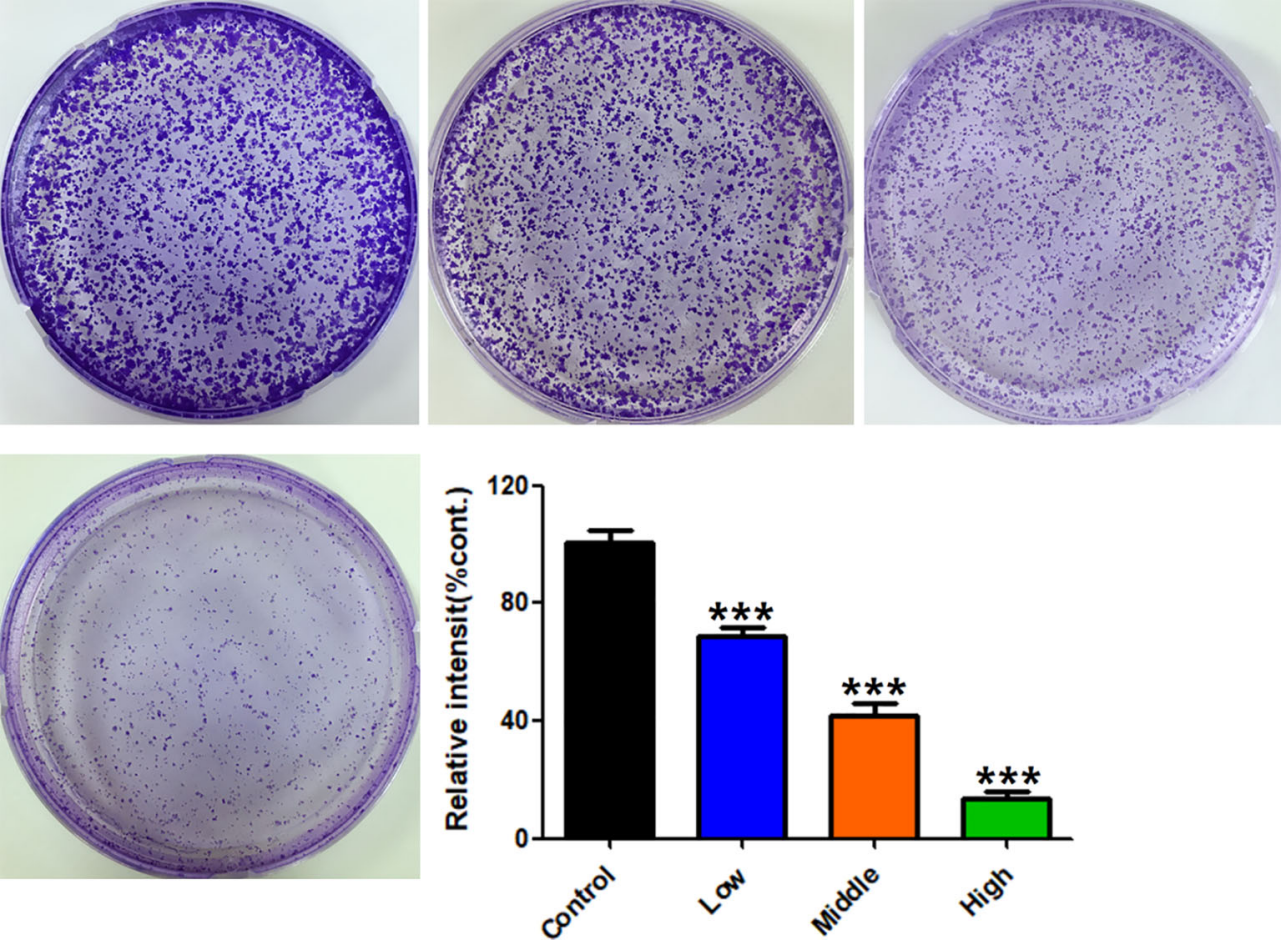
Effects of RPL on cloning formation of A549 cells. Cells were treated with RPL (0.2, 0.4, and 0.6 mg/mL) for 14 days and then stained with crystal violet. The colony formation was observed under a microscope, and five fields were randomly selected for counting. Data were expressed as mean ± SD (n = 3), and asterisk indicated significant difference, ***p < 0.001 vs. control.

Consistent with CCK-8 results, RPL significantly inhibited the cloning formation of A549 cells compared with the control cells (**Figure 8**), and the inhibition was concentration-dependent (0.2, 0.4, and 0.6 mg/mL). Taken together, these results suggest that RPL could inhibit the proliferation of A549 cells.

### RPL Induces Aapoptosis in A549 Cells

**Figure 9:**
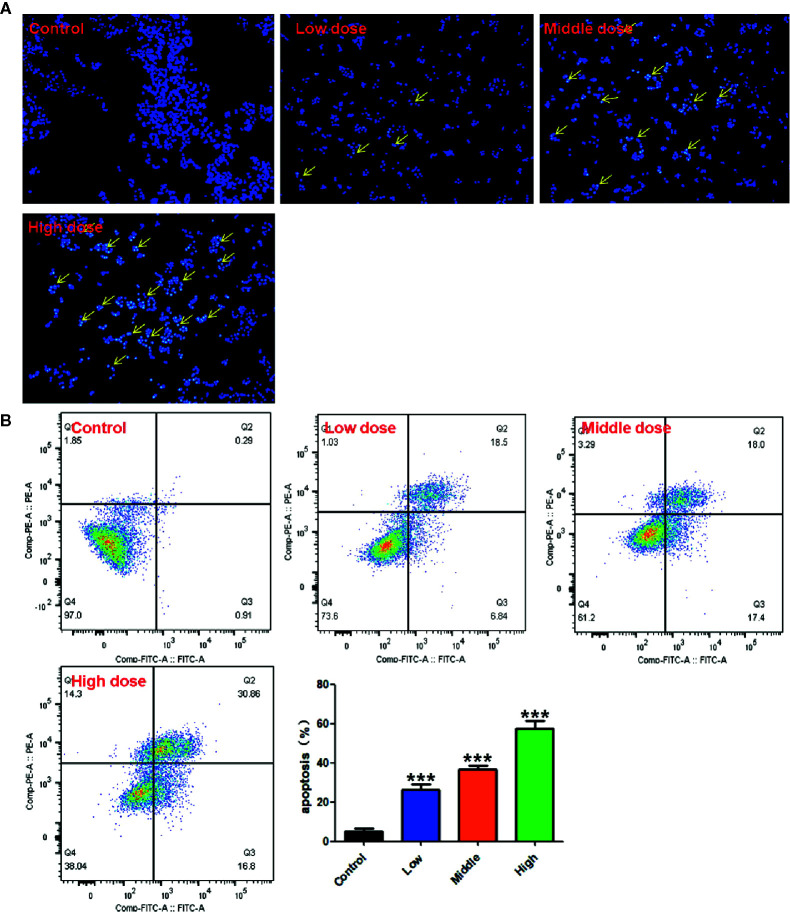
Apoptotic induction acitivities of RPL on A549 cells. Cells were treated with RPL (0.2, 0.4, and 0.6 mg/mL) for 24 h, and the apoptotic effects of RPL were detected by **(A)** DAPI staining assay with a fluorescence microscope and **(B)** flow cytometric analysis with Annexin V-FITC and PI staining. ***p < 0.001 vs. control.

Previous results suggested that RPL had a significant anti-proliferation effect on A549 cells. To determine whether this anti-proliferation effect was related to apoptosis, DAPI staining and flow cytometry were used to detect the apoptosis-inducing effect of RPL on A549 cells. As shown in **Figure 9A**, the experimental results showed that weak fluorescence could be observed in the control A549 cells (intact nucleus) after DAPI staining. On the contrary, A549 cells treated with RPL (0.2, 0.4, and 0.6 mg/mL) showed significantly increased fluorescence brightness, suggesting that the nucleus underwent morphological changes of apoptosis, such as nuclear condensation and nuclear shrinkage. Subsequent flow cytometric analysis further confirmed the pro-apoptotic effect of RPL. As can be seen from the **Figure 9B**, with the increase of RPL concentration ranging from 0.2 to 0.6 mg/mL, the apoptosis rate of A549 cells increased gradually (P < 0.01, P < 0.01, P < 0.01, respectively, vs. untreated A549 cells). These results suggest that RPL can induce apoptosis of human lung cancer cells.

### Monmers in RPL Possess Cytotoxic Effects on A549 Cells *via* Induction of Apoptosis

**Figure 10:**
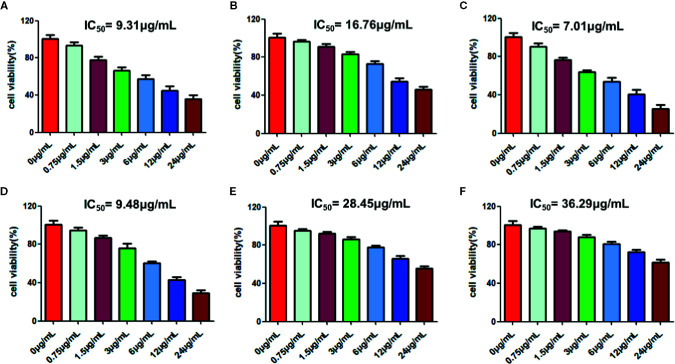
Cytotoxic effects of emodin **(A)**, resveratrol **(B)**, aloe-emodin **(C)**, rhein **(D)**, chrysophanol **(E)**, and physcion **(F)** on A549 cells. Cells were treated with the candidate drugs (0, 0.75, 1.5, 3.0, 6.0, 12, and 24 µg/mL) for 24 h. Finally, CCK-8 assays were carried out to detect the cytotoxic effects of candidate drugs. Each experiment was repeated three times individually. The IC_50_ values were calculated for evauation of the cytotoxic effects of candidate drugs.

Furthermore, we studied the cototoxic effects of some avialiable monmers in RPL on the lung cancer cell line of A549, and our present results showed that these monmers in RPL including emodin, resveratrol, aloe-emodin, rhein, chrysophanol, and physcion show significant inhibitory effects on the proliferation of A549 cells with the IC_50_ values below 40μg/mL (9.31, 16.76, 7.01, 9.48, 28.45, and 36.29 μg/mL for emodin, resveratrol, aloe-emodin, rhein, chrysophanol, and physcion, respectively) (**Figure 10**).

**Figure 11:**
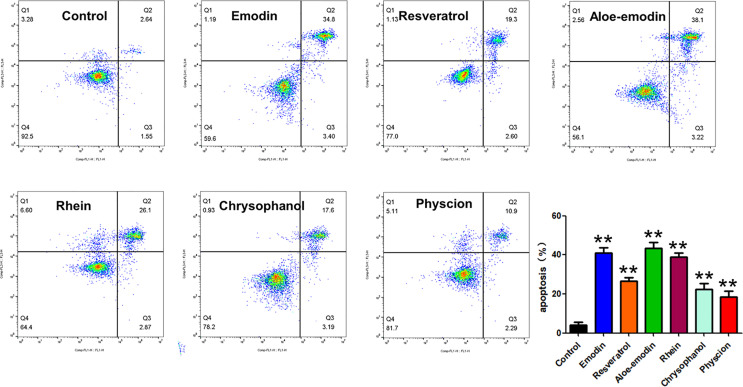
Apoptotic induction activities of emodin, resveratrol, aloe-emodin, rhein, chrysophanol, and physcion on A549 cells. Cells were treated with candidate drugs (6 µg/mL) for 24 h, and the apoptotic cells were detected by staining with Annexin V-FITC/PI followed by flow cytometry analysis. Data were expressed as mean ± SD (n = 3), and asterisk indicated significant difference, **p < 0.01 vs. control.

Then, we also investigated whether the anti-proliferative effects of these monmers are related to indcution of apoptosis or not. Interesting, our results suggested that all the testing monmers from RPL showed obvious pro-apoptic potentials on A549 cells (**Figure 11**).

## Discussion

It’s no doubt that TCMs can be used to prevent or treat various difficult miscellaneous diseases (Newman and Cragg, 2016; Long et al., 2020), and also promising resources for discovering more candidate drugs for treating tumors. Due to the TCMs are characterized as “multi-components”, “multi-targets,” and “multi-approaches,” it would take a lot of manpower, material resources, and time to study the effects and mechanisms of herbal medicines by routine pharmacological experiments, which is also the big gap for wide acceptance and use of herbal medicine in clinic.

Network pharmacology enables rapid screening of drugs and targets, as well as analysis of pathways of action, greatly reducing the time and cost of drug development. In addition, network pharmacology can systematically analyze the interaction between drugs and diseases, which is basically consistent with the overall concept of TCMs. Meanwhile, it also provides new ideas and technical means to study the material basis and mechanism of action of TCMs. In this study, network pharmacology was used to study the potential active compounds and possible targets of RPL in the treatment of lung cancer, combined with *in vitro* experiments for verification, to scientifically and systematically clarify the material basis and mechanism of RPL in the treatment of lung cancer.

RPL is a famous TCM which has been used for thousands of years with the functions of “*attacking stagnation, clearing damp and heat, purging fire, cooling blood, removing stasis and detoxifying*” and is currently used for treating various diseases, such as high fever, constipation, abdominal pain, and tumor, etc (Aichner and Ganzera, 2015; Liu et al., 2016). In our present results, 20 potential compounds from RPL were screened, and interestingly these 20 compounds have appeared in the drug ingredients and disease targets (DDN), indicating that anti-tumor effects of RPL might be closely related to these 20 compounds mentioned above, including one terpenoids, two steroids, six flavonoids, and seven anthraquinones. According to the degree of each compound in DDN, the compounds numbered C2 (Torachrysone-8-O-β-D-(6’-oxayl)-glucoside), C6 (Procyanidin B-5,3’-O-gallate), C9 (Mutatochrome), C10 (gallic acid-3-O-(6’-O-galloyl)-glucoside), C15 (Aloe-emodin), C17 (Emodin), C18 (Resveratrol), C19 (Chrysophanol), and C20 (Physcion) are considered as the main active components of RPL for anti-lung cancer. Some of these mentioned compounds, such as aloe-emodin, emodin, and resveratrol have been reported to can be used for treating lung cancer (Peng et al., 2013; Dong et al., 2016; Dong et al., 2020). In addition, Mao *et al.* reported that Procyanidins can play an anti-lung cancer activity by inhibiting inflammation and inducing tumor cell apoptosis, and the mechanism of action is related to the inhibition of cyclooxygenase-2 (COX-2)/prostaglandin E2 (PGE2) eicosan-like pathway and the initiation of caspase-3 apoptosis pathway, suggesting that Procyanidins can be used as a potential preventive and therapeutic drug against lung cancer, which has been also supported by a recent clinical study (Mao et al., 2016; Mao et al., 2019).

Furthermore, our results also suggest that INS, AKT1, EGFR and TP53 are the key targets of anticancer activities of active compounds in RPL. INS, which encoded insulin in the body, plays a crucial role in regulating carbohydrate and lipid metabolism. Growing reports have suggested that insulin may increase the risk of certain cancers, such as prostate, rectal, breast and pancreatic cancers (Hsing et al., 2001; Goodwin et al., 2002; Stolzenberg-Solomon et al., 2005; Limburg et al., 2006; Albanes et al., 2009). A case cohort study in 2017 found that higher insulin levels and insulin resistance may increase the risk of lung cancer (Argirion et al., 2017). In addition, Hayakawa et al. found that patients with non-small cell lung cancer (NSCLC) would further develop resistance to the available treatments by activating insulin-like growth factor-1 receptor (IGF1R) (Hayakawa et al., 2020). In addition, it’s reported that INS plays an important role in the proliferation and metastasis of NSCLC cells (Li S. et al., 2019). AKT is an important kinase in the PI3k-AKT pathway, which is catalyzed by PI3K to migrate to the cell membrane and activate (Zhang et al., 2019). Activated Akt regulates the proliferation and growth of cells and is also involved in the apoptosis and glucose metabolism. In addition, insulin also activates the Akt signaling pathway to conservatively execute the insulin metabolic response in tumor cells, driving the metabolism of tumor cells. Thus, AKT has become an attractive intervention target for the development of antitumor drugs. Alpha subunit protein kinase B (AKT1) is an important member of the AKT family, and current studies have confirmed that AKT1, as an important regulatory factor, is in a state of disorder in various tumors, such as prostate cancer, osteosarcoma, ovarian cancer, and endometrial cancer, leading to uncontrolled proliferation of tumor cells, apoptosis defects, and enhanced metastasis and invasion ability of tumor (Alwhaibi et al., 2019; Huo et al., 2019; Wang J. et al., 2019). Rao et al. found that AKT1 is a key metastasis regulator in NSCLC cells, and *in vitro* inhibition of AKT1 can promote invasion and metastasis of NSCLC cells with K-RAS or EGFR mutations (Rao et al., 2017). It has been reported that long non-coding RNAs can inhibit the proliferation and invasion of NSCLC cells by targeting the AKT1 signaling pathway (Zhang et al., 2018). Another study also found that MicroRNAs can inhibit cell proliferation, migration and invasion of lung cancer, regulate cell cycle distribution and epithelial-mesenchymal transition (EMT), the mechanism of which is related to down-regulation of AKT1 expression (Wu et al., 2019). Epidermal growth factor receptor (EGFR) is a glycoprotein that plays an important role in physiological processes such as proliferation, apoptosis and differentiation of cell by activating the calcium signaling pathway, MAPK signaling pathway and PI3K/AKT signaling pathway (Carlisle et al., 2007). As an important anticancer gene, the inactivation of p53 plays an important role in tumor formation, and increasing studies have found that the p53 plays a crucial role in retarding tumor cell cycle, promoting tumor cell apoptosis and inhibiting tumor angiogenesis (Bi et al., 2018; Li P. et al., 2019; Wang et al., 2020).

For analysis of the GO function annotation of the target genes, it revealed that the related target cell components for anticancer activity of this herbal medicine include nucleus cytoplasm, exosomes, cytosol, lysosome, and extracellular region; its biological processes are involved in metabolism, energy pathways, apoptosis, neurotransmitter metabolism, and protein metabolism and others; and the related molecular functions are associated with catalytic activity, protein serine/threonine kinase activity, ligand-dependent nuclear receptor activity, cysteine-type peptidase activity, and carboxy-lyase activity. What’s more, KEGG pathway analysis revealed these genes were significantly enriched in a variety of cancer pathways, nitrogen metabolism, neurotrophin signaling pathway, EGFR tyrosine kinase inhibitor resistance, platinum drug resistance, and apoptosis pathways.

Collectively, the GO and KEGG pathway analysis suggested that induction of apoptosis appeared to be a potential way for the anticancer activities of RPL against lung cancer. Abnormal proliferation and insufficient apoptosis in tumor cells are the main causes of lung cancer. Therefore, inhibiting the proliferation and inducing apoptosis of lung cancer has become a feasible strategy for the treatment of lung cancer. A large number of studies have also found that mechanisms of many natural anticancer drugs against lung cancer are related to the regulation of different signaling pathways to inhibit the proliferation of lung cancer cells and induce the apoptosis of lung cancer cells (Wang S. et al., 2019; Han et al., 2020; Hu et al., 2020). Therefore, we subsequently designed corresponding *in vitro* experiments in order to prove the prediction results of network pharmacology. CCK-8 results showed that RPL could inhibit the proliferation of A549 cells with a concentration-dependent manner. Furthermore, we carried out the CCK-8 assays on a normal human cell line of HUVECs to evaluate the toxicological effect RPL. Importantly, RPL does not appear to inhibit the growth of normal HUVEC cells. Furthermore, RPL treatment can also reduce the clone formation rate of A549 cells, further proving that this TCM can inhibit the proliferation ability of A549 cells. The results of DAPI staining and flow cytometry results showed that the anti-proliferation effect of RPL on lung cancer cells was related to the induction of apoptosis. Furthermore, we also determined the apoptotic effects of some characteristic constituents in RPL against lung cancer cells of A549, including emodin, resveratrol, aloe-emodin, rhein, chrysophanol, and physcion. From the results of CCK-8 and flow cytometry assays, it’s showed that all of the 6 monomers exhibited the IC_50_ values below 50μg/mL, and could induce significant apoptosis in A549 cells. All these experimental results confirmed the prediction results of network pharmacology.

Based on the experimental evidences of previous works and our studies, it’s suggested that RPL can play an anticancer role by inducing apoptosis of lung cancers. However, some works are also needed to be devoted in the further mechanisms of this plant for anti-lung cancers *via* induction of apoptosis. In addition, we also reported some active constituents with anti-lung cancer properties in this plant, however the *in vivo* experiments for confirming their activities are also necessary. Besides, from the results of our study, it’s also suggested that the application of network pharmacology to the study of TCM is a scientific and feasible method.

## Conclusion

This study is the first to apply network pharmacology method to study the material basis and action mechanism of RPL against lung cancer. The results showed that 16 active ingredients in RPL had potential anti-lung cancer activity, involving 563 target genes related to lung cancer. Our results showed that C2 (Torachrysone-8-O-β-D-(6’-oxayl)-glucoside), C6 (Procyanidin B-5,3’-O-gallate), C9 (Mutatochrome), C10 (gallic acid-3-O-(6’-O-galloyl)-glucoside), C15 (Aloe-emodin), C17 (Emodin), C18 (Resveratrol), C19 (Chrysophanol), and C20 (Physcion) are the main active components of RPL in the treatment of lung cancer. INS, AKT1, EGFR, and TP53 are the hub genes of RPL in the treatment of lung cancer. The mechanism of RPL against lung cancer is related to 134 signaling pathways, and the key mechanism of RPL against lung cancer may be related to inducing lung cancer cell apoptosis. These network pharmacological predictions were verified in subsequent cell experiments. This study studied the pharmacodynamic basis and mechanism of RPL on lung cancer from the perspective of systematic pharmacology, providing a scientific basis for further elucidating the effect of rhubarb on lung cancer.

## Data Availability Statement

The datasets generated for this study are available on request to the corresponding authors.

## Author Contributions

All authors contributed to the article and approved the submitted version. CW and WJ conceived and designed this paper. QZ, JL, RL, RZ, and DR completed the experiments. MZ and SW analyzed the data. QZ and JL wrote the original draft of the paper. CW reviewed and edited the paper.

## Conflict of Interest

The authors declare that the research was conducted in the absence of any commercial or financial relationships that could be construed as a potential conflict of interest.

## Abbreviations

RPL, *Rheum palmatum* L.; TCM, traditional Chinese medicine; OB, Oral bioavailability; DL, Drug-Likeness; DDN, drug ingredients and disease targets; PPI, Protein-Protein Interaction Network; GO, gene ontology; KEGG, Kyoto Encyclopedia of Genes and Genomes; CC, cellular component; BP, biological process; MF, molecular function; FBS, Fetal bovine serum; PBS, phosphate buffered saline; DMEM, dulbecco modified eagle medium; DMSO, Dimethyl sulfoxide; DAPI, 4′,6-diamidino2- phenylindole; CCK8, Cell Counting Kit-8; HUVEC, human umbilical vein endothelial cells; OD, optical density; SD, standard deviation; ANOVA, one-way analysis of variance; COX-2, cyclooxygenase-2; PGE2, prostaglandin E2; NSCLC, non-small cell lung cancer; IGF1R, insulin-like growth factor-1 receptor; EMT, epithelial-mesenchymal transition; EGFR, Epidermal growth factor receptor.
