# Supplementary material for: A Network Pharmacology Approach to Investigate the Anticancer Mechanism and Potential Active Ingredients of Rheum palmatum L. Against Lung Cancer via Induction of Apoptosis
Source: Front Pharmacol. 2020 Nov 4;11:528308. doi: 10.3389/fphar.2020.528308 (PMC7672213; doi:10.3389/fphar.2020.528308)
Supplement: Supplementary file 1 [file DataSheet_1.docx]

**A network pharmacology approach to investigate the anti-cancer mechanism and potential active ingredients of *Rheum palmatum* L against lung cancer via induction of apoptosis**

Qing Zhang^1,#^, Jia Liu^1,#^, Ruolan Li^1^, Rong Zhao^1^, Mengmeng Zhang^1^, Shujun Wei^2^, Dong Ran^1^, Wei Jin^2,*^, Chunjie Wu^1*^

^1^School of Pharmacy, Chengdu University of Traditional Chinese Medicine, Chengdu 611137, P.R. China;

^2^Emergency Department, Hospital of Chengdu University of Traditional Chinese Medicine, Chengdu 610072, P.R. China;

*Correspondence author: Wei Jin

Correspondence address: Emergency Department, Hospital of Chengdu University of Traditional Chinese Medicine, No. 39, Shi-er-qiao Road, Chengdu 610072, P.R. China;

Phone and Fax: +86-028-87783481;

E-mail: jinwei1983@cdutcm.edu.cn (W.J);

*Correspondence author: Chunjie Wu

Corresponding address: School of Pharmacy, Chengdu University of Traditional Chinese Medicine, No. 1166, Liutai Avenue, Chengdu 611130, P.R. China;

Phone and Fax: +86-028-61801001

E-mail: wucjcdtcm@163.com (C.J.W);

^#^ Qing Zhang and Jia Liu contributed equally to this paper.

***HPLC assay***

HPLC assay was performed on an SHIMADZU LC-20A HPLC system (Tokyo, Japan) with a Thermo Hypersil ODS C_18_ chromatographic column (250 mm × 4.6 mm, i.d. 5 μm, Shiseido, Japan) at 254 nm, sample injection volume was 10 μL, and column temperature was set at 40 ºC. Separation was performed using gradient elution (acetonitrile (A) /0.1% aqueous phosphoric acid (B)) gradient at a flow rate of 1.0 mL/min. Samples were analyzed by using a gradient program as follows: 0 - 10 min, 95 - 85 % B; 10 - 50 min, 85% B; 507 - 60 min, 85 - 80% B; 60 - 75 min, 80 - 75% B; 75 - 90 min, 75% B; 90 - 100 min, 75 - 40% B; 100 - 125 min, 40 - 15% B; 125 - 130 min, 15 - 0% B. All the compounds were identified with the retention time and reference substances [purchased from the PUSH Bio-Technology Co. (<https://www.push-herbchem.com/>, Chengdu, China) with the purity over 98%].

***Result***

The HPLC assay of the RPL extracts was carried out and the results showed the main constituents in RPL are anthraquinones such as Aloe-emodin-8-O-glucopyranoside, Rhein-8-O-glucopyranoside, Chrysophanol-1-O-glucopyranoside, Chrysophanol-8-O- glucopyranoside, Aloe-emodin, Emodin, Chrysophanol, *etc* (Figure S1).


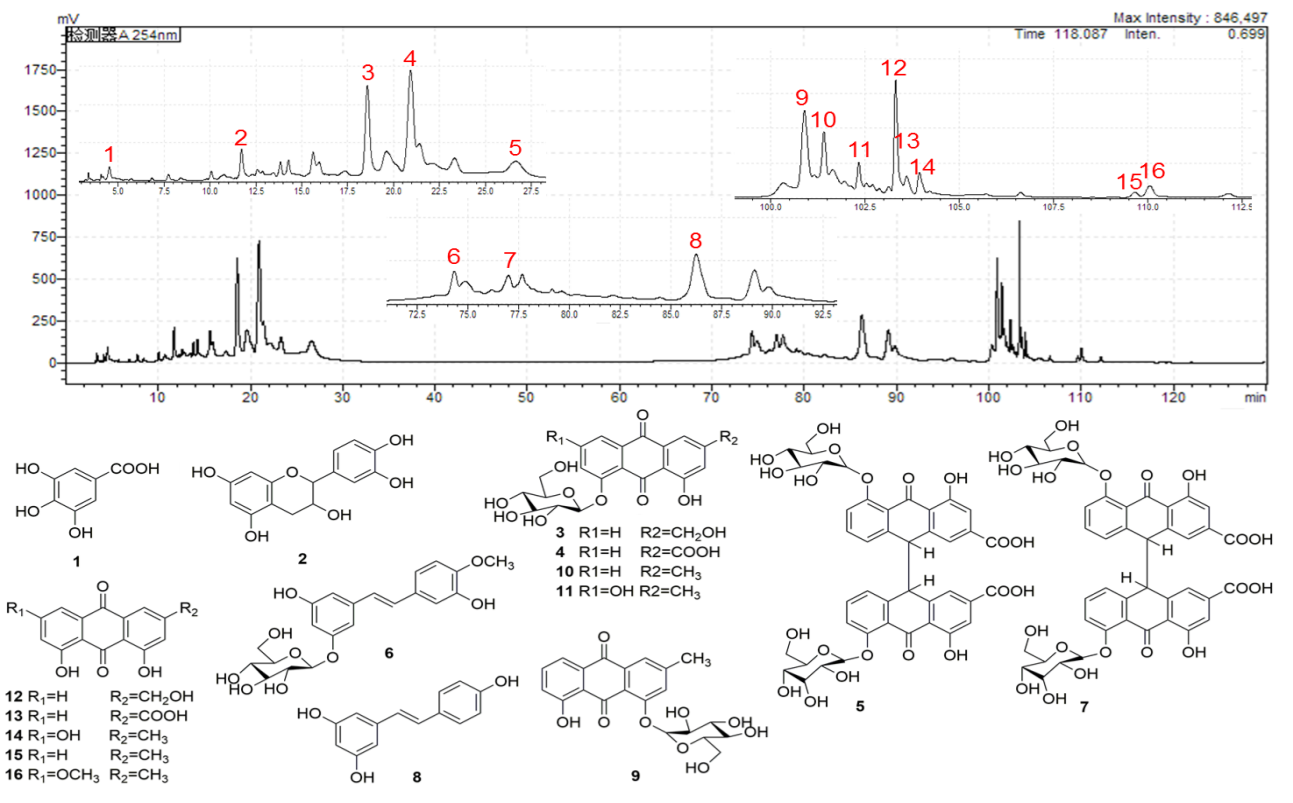


Figure S1. The HPLC chromatograms of RPL extracts.

Gallic acid (1), Catechin (2), Aloe-emodin-8-*O*-β-*D*-glucopyranoside (3), Rhein-8-*O*-β-*D*-glucopyranoside (4), Sennoside B (5), Rhaponticin (6), Sennoside A (7), Resveratrol (8), Chrysophanol-1-*O*-β-*D*-glucopyranoside (9), Chrysophanol-8-*O*-β-*D*-glucopyranoside (10), Emodin-8-*O*-β-*D*-glucoside (11), Aloe-emodin (12), Rhein (13), Emodin (14), Chrysophanol (15), Physcion (16).
